# On the importance of simultaneous label-free multimodal nonlinear optical imaging for biomedical applications

**DOI:** 10.1063/5.0289864

**Published:** 2025-11-18

**Authors:** Alejandro De la Cadena, Jaena Park, Jindou Shi, Stephen A. Boppart

**Affiliations:** 1 Beckman Institute for Advanced Science and Technology, University of Illinois Urbana-Champaign, Urbana, Illinois 61801, USA; 2 Department of Bioengineering, University of Illinois Urbana-Champaign, Urbana, Illinois 61801, USA; 3 Department of Electrical and Computer Engineering, University of Illinois Urbana-Champaign, Urbana, Illinois 61801, USA; 4 NIH/NIBIB Center for Label-free Imaging and Multiscale Biophotonics, University of Illinois Urbana-Champaign, Urbana, Illinois 61801, USA

## Abstract

Label-free nonlinear microscopy offers a powerful tool for the biomedical sciences. It enables investigations of cells and tissues using signals that emerge from endogenous biomolecules and microstructures to derive contrast, thereby preserving the physiological viability and functionality of specimens. Today, the most advanced label-free nonlinear microscopes are multimodal imaging platforms that capitalize on the heterogeneity of biological specimens, capturing not one but many nonlinear signals. Thus, label-free multimodal nonlinear imaging attains a contrast palette with complementary signals, delivering data-rich images that not only allow spatial unmixing and quantification of biochemical species but also unleash the power of correlation analyses and artificial intelligence to extract further information from specimens. In this Perspective, we recap the nonlinear contrast palette and compare the two technological strategies often used to acquire multimodal nonlinear images: a sequential approach vs a simultaneous approach. We then present their strengths and weaknesses and discuss emerging computational strategies that enhance the interpretability of multimodal data.

## I. INTRODUCTION

**FIG. 1.**
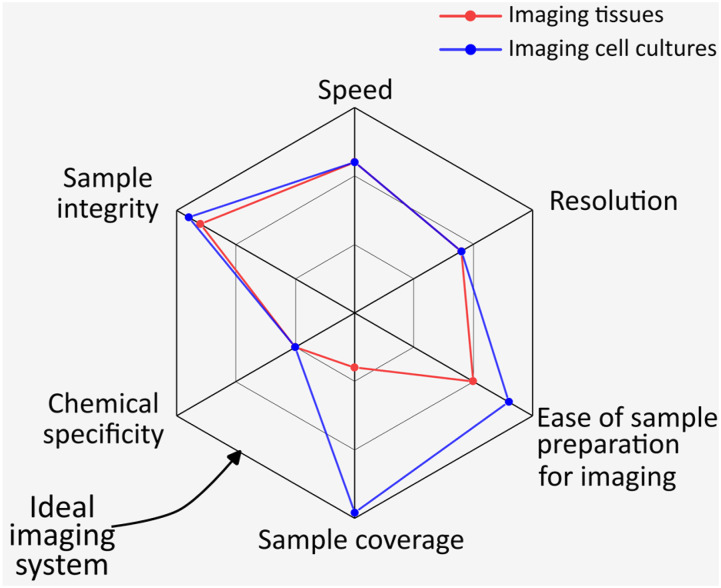
Strengths of multimodal label-free nonlinear imaging, an approach that comes close to the ideal imaging system.

Nonlinear light–matter interactions drive a plethora of optical signals that inform about the physicochemical properties of a material.^1–3^ When coupled with high-numerical-aperture objectives, these nonlinear signals derive imaging contrasts that expose both architecture and chemical constitution.^4^ The intrinsic nonlinear signals originating from species native to biological systems allow label-free imaging,^5^ a capability that averts the use of exogenous labels, which, albeit highly specific and outstandingly robust, can be larger than their targets and may perturb the natural order and behavior within the cellular milieu.^6^ By imaging life, not labels, label-free imaging preserves the physiological viability of cells and tissues, reduces the steps required for sample preparation, supports high-speed imaging with subcellular resolution and optical sectioning, and allows real-time investigations *in vivo.*^7,8^ Owing to their heterogeneity, biological specimens mediate not a few but many nonlinear contrasts,^9–12^ enabling yet another benefit of label-free nonlinear microscopy, namely, multimodality, a feature that confers chemical specificity to the nonlinear microscope. Figure 1 summarizes the strengths of multimodal label-free imaging, qualitatively comparing a hypothetically ideal imaging system (black trace) with state-of-the-art microscopes mapping cells (blue curve) or tissues (red curve).

The first application of label-free nonlinear microscopy for mapping a biological specimen was reported over four decades ago by Duncan *et al.*^13^ and widely popularized by Webb and colleagues^14^ over three decades ago. Common to both was the derivation of contrast from specimens using only one nonlinear signal, with the earliest implementation focusing on coherent anti-Stokes Raman scattering (CARS) and the latter on two-photon absorption fluorescence (2PAF). Today, the apex of the technological progress in label-free nonlinear microscopy is multimodal imaging, i.e., the integration of multiple nonlinear signals into a unified contrast palette.^15–21^ Because each nonlinear signal offers unique advantages and limitations, a rich contrast palette adds complementarity to the nonlinear microscope, thereby enhancing its robustness and analytical power. Since each nonlinear signal targets a specific species, a multimodal approach enables the spatial separation of different constituents within the sample by virtue of their spectroscopic properties,^22^ a separation that supports both the localization and quantification of biochemical and microstructural constituents.^23–27^ Furthermore, the nonlinear microscope can be further strengthened by linear techniques sensitive to structural features of specimens, such as quantitative phase imaging or optical coherence microscopy.^21^

Beyond spatial unmixing and quantification, label-free multimodal imaging also reveals co-localization, indicating whether distinct molecular species or microstructures occupy the same physical 3D space within a sample. In the context of cells and tissues, co-localization may suggest potential interactions between different species, co-regulation, or their association with the same structures.^28^ This spatial co-localization can indicate, for example, where structural and chemical features overlap in diseased tissue. In addition to spatial overlap, multimodal systems support correlation analysis, which quantifies how signals from different channels vary together across the image.^29^ Label-free multimodal imaging then can uncover the functional relationships between components of the sample, even when they do not co-localize, exposing the coordinated changes between the channels over different regions of a specimen that may relate to shared biological processes. Therefore, both co-localization and correlation analyses offer a clear, interpretable, and hypothesis-driven glimpse into spatial and statistical relationships between nonlinear signals.^30^

Label-free multimodal imaging is further strengthened by artificial intelligence (AI), which maximizes its ability to extract information derived from subtle details and patterns within the images that are frequently imperceptible to human judgment.^31,32^ Through feature extraction strategies, deep learning (DL) can identify shapes, intensity profiles, textures, and spatial context across imaging channels. Since each nonlinear contrast contributes a distinct set of features, these networks can uncover “hidden” patterns, patterns that may reflect both dynamic and static morphofunctional properties of specimens. Therefore, the more nonlinear contrasts, the more dimensions available to improve pattern recognition, driving downstream tasks, such as classification, segmentation, restoration, and data-driven discovery.

Finally, label-free multimodal imaging operating in a multiplex configuration—where all contrasts are acquired simultaneously and in parallel—not only secures strict spatial co-registration, but also temporal co-registration, eliminating artifacts derived from mechanical vibrations or due to time-dependent dynamics within specimens. Unlike sequential acquisition approaches, which can be slower and prone to temporal misalignment, multiplex detection accelerates imaging speed, making label-free multimodal spatiotemporal imaging more suitable for capturing biological processes.

**FIG. 2.**
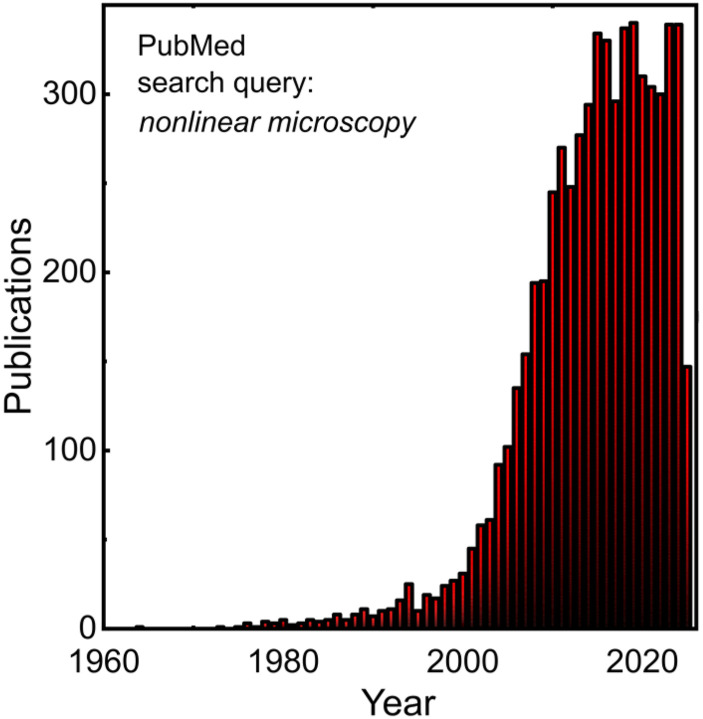
Publication trend for nonlinear microscopy. Note that this search is general, and includes all developments within the field, which also reflect the progress of label-free imaging.

Manifestly, label-free multimodal nonlinear microscopy offers a manifold of benefits to a wide range of scientific disciplines, most notably biology and medicine. By probing endogenous molecules, it delivers a robust contrast palette with complementary signals, unmixes, and quantifies biochemical constituents, supports statistical analysis, and facilitates the discovery of hidden patterns within datasets. A testament to the impact and potential of this technology is the consistent and growing number of publications and research outputs employing this technology (Fig. 2) and the recognition and emergence of U.S. federal support from the National Institutes of Health’s National Institute for Biomedical Imaging and Bioengineering’s Center for Label-free Imaging and Multiscale Biophotonics (CLIMB).^33^ This success stems from technological innovations, combined with clever engineering design.

Our group has contributed to and pioneered several of these developments, accumulating a repository of data and empirical knowledge for over a quarter of a century. In this Perspective, we draw from this archive to reflect on the evolution, current capabilities, and future directions of label-free multimodal nonlinear microscopy. We begin by outlining the nonlinear contrast palette, giving an emphasis on the biological targets of these nonlinear signals (Sec. II). We then highlight the key innovations that have enabled the field to evolve from narrowband acquisition toward multiplex detection (Sec. III), describing the pros and cons of simultaneous vs sequential imaging in multiplex platforms. We then discuss emerging computational strategies—co-localization, machine, and deep learning—that enhance the interpretability and analytical power of multimodal data (Sec. IV). Finally, we conclude (Sec. V) by considering future directions.

## II. THE NONLINEAR CONTRASTS

The nonlinear signals most commonly used for imaging are the second and third harmonic generation (SHG and THG) signals, multiphoton absorption fluorescence (MPAF) intensity and lifetime, and coherent Raman scattering (CRS). All of these signals originate from light–matter interactions, usually involving ultrashort femtosecond (fs) pulses to elicit a response of the specimen. Here, we briefly describe these contrasts and their biochemical targets.

### A. Second harmonic generation (SHG)

SHG is a nonlinear optical phenomenon, in which a fundamental light field oscillating at frequency $\omega_{1}$ gives rise to a second optical field at $2\omega_{1}$. In this process, two photons of the fundamental field interact with the material, undergo annihilation, and simultaneously generate a single photon with frequency $\omega_{2}=2\omega_{1}$. Consequently, the SHG field has half the wavelength of the fundamental. SHG is a second-order nonlinear process mediated by $\chi^{\left(2\right)}$ (the second-order nonlinear susceptibility), and since not all materials possess a non-zero $\chi^{\left(2\right)}$, the SHG process is highly selective for non-centrosymmetric structures. Because SHG does not involve real electronic transitions, there is no net energy transfer from the field to the material system, rendering SHG immune from photobleaching.

SHG offers to biosciences intrinsic selectivity toward non-centrosymmetric molecular or structural assemblies. In microscopy, SHG enables the visualization of highly ordered macromolecular structures, most notably fibrillar collagen, exposing the organization and composition of the tissue microenvironment.^34–39^ Endogenous SHG targets include protein arrays, such as microtubule bundles (tubulin polymers), actomyosin networks, and collagen fibers. By imaging these non-centrosymmetric constituents, SHG microscopy provides a reliable tool to assess tissue architecture and its dynamic alterations in response to pathological conditions.

### B. Third harmonic generation (THG)

THG arises from the simultaneous interaction of three photons at a fundamental frequency $\omega_{1}$, resulting in the emission of a single photon at the third harmonic frequency $\omega_{2}=3\omega_{1}$. Like SHG, THG is a parametric process, meaning there is no net energy exchange between the optical fields and the medium. However, unlike SHG, THG originates from the third-order nonlinear susceptibility $\chi^{\left(3\right)}$. Consequently, THG can occur in all media, including isotropic and centrosymmetric biological specimens.

THG is sensitive to interfaces, structural inhomogeneities, and subcellular features, making it well-suited for imaging transparent and heterogeneous specimens, such as cells and tissues. While THG lacks intrinsic chemical specificity, it can reveal lipid-rich regions, trace individual lipidic bodies, and map the spatial distribution of fatty structures because of the large refractive index difference across lipid–aqueous interfaces—capabilities that earn it a distinct role within the nonlinear contrast palette for studying biological systems.^40–48^ Its ability to image nanometric features also makes this contrast particularly promising for cancer research, where it has been used to identify and track extracellular vesicles involved in cellular communication and signaling.^17,49^

### C. Multiphoton absorption fluorescence

**FIG. 3.**
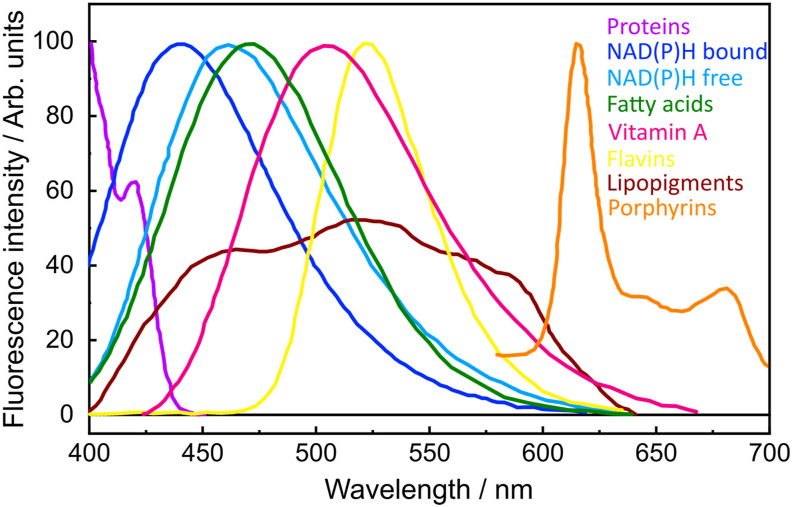
Spectral profiles of autofluorescence emission from single endogenous fluorophores. Adapted from Ref. 12.

Multiphoton absorption refers to a nonlinear light–matter interaction, in which an electronic excited state of a molecule is accessed through the simultaneous absorption of two or more photons. Multiphoton absorption fluorescence (MPAF) microscopy exploits the emissive intensity and decay lifetime of molecules excited in this way to derive contrast. Endogenous molecules with strong MPAF are the metabolic cofactors NAD(P)H and FAD,^50^ species that partake in cellular redox reactions and, through their emissive nature, inform about metabolic states.^12^ Another group of important autofluorescent species are lipopigments, most notably lipofuscin, which accumulates in response to oxidative stress and cellular aging, thereby providing intrinsic contrast in aged or diseased tissues. In addition, MPAF from amyloid-beta deposits—a pathological hallmark of Alzheimer’s disease—has been recently studied as a label-free marker, enabling visualization of pathology in its native and unaltered form.^51,52^ These endogenous signals enable nondestructive, chemically specific imaging without the need for external dyes, probes, or nanoparticles, making MPAF a valuable tool for *in vivo* studies, longitudinal imaging, and applications in developmental biology, cancer research, and neuroscience. In fact, two forms of MPAF—two-photon absorption fluorescence (2PAF) and three-photon absorption fluorescence (3PAF)—are backbones of the nonlinear contrast palette. Figure 3 shows the emission spectra of endogenous fluorophores.

### D. Coherent Raman scattering (CRS)

Owing to their high sensitivity and chemical specificity toward molecular vibrations, coherent Raman techniques—particularly coherent anti-Stokes Raman scattering (CARS) and stimulated Raman scattering (SRS)—have been applied in the investigation of cells, model organisms, and tissue specimens.^53–57^ CARS and SRS have shown potential in lipid biology,^58–62^ enabling visualization of lipid droplets and revealing their dynamics and compositional heterogeneity. These modalities have also been used in the study of,exposing the molecular orientation and vibrational signatures of the myelin sheath in the spinal cord.^63,64^ Beyond the research laboratory, the diagnostic potential of coherent Raman microscopy has extended into clinical settings, where it has been used both to validate tumor diagnoses against conventional hematoxylin and eosin (H&E) staining and to provide real-time intraoperative imaging in surgical oncology.^65–70^

**FIG. 4.**
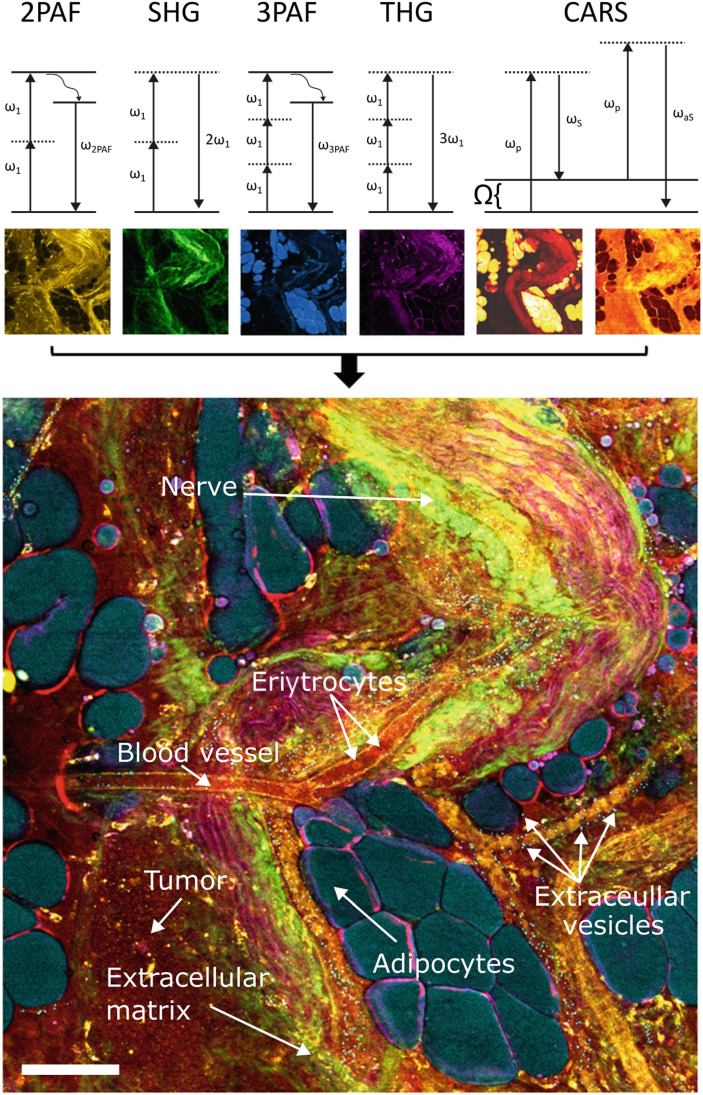
Nonlinear signals and their potential for imaging. The top row shows the energy diagrams of the nonlinear signals used to derive label-free contrast, while the image below depicts a rat mammary tumor (lower left corner) and the microenvironment painted with the nonlinear contrast palette. Scale bar: 80 *µ*m. Image adapted from Ref. 83.

Table I summarizes the nonlinear signals used to derive contrast in nonlinear microscopy, along with their endogenous targets, while Fig. 4 shows an exemplar image using the nonlinear contrast palette. It is worth noting that these are just one set of nonlinear signals useful to investigate biological specimens. There are other relevant nonlinear signals for label-free imaging, most notably imaging based on pump–probe^71–81^ and sum-frequency generation.^82^ However, because they have not been widely integrated with other contrasts, they are not discussed further.

**TABLE I.** Signals of the nonlinear contrast palette and their endogeneous targets.

Nonlinear signal	Target biochemical species
SHG	Collagen, actomyosin, and microtubules
THG	Lipids, membranes, interfaces, extracellular vesicles, and nerve fibers
MPAF (2PAF, 3PAF)	Autofluorescent molecules [(NAD(P)H, riboflavin (FAD), amyloid-β deposits, tryptophan, melanin, retinol, and lipofuscin]
CRS (CARS, SRS)	Lipids, myelin, proteins (CH, CH_2_, and CH_3_ modes), nucleic acids, and neurotransmitters

It is worth mentioning that the fact that multiple, but different, biochemical species coexist at the same location in the specimen is not in itself an issue. This phenomenon is in fact often encountered—consider, for example, tissues packed with adipocytes, cells that produce strong THG, fluorescence, and CRS. If we consider each image from a particular contrast as a realization of a stochastic process, the overall intensity distribution, or histogram, of each channel should be unique. It is the difference between the (probability) distribution of each channel that makes multimodal label-free imaging a powerful technique, as it not only allows the extraction of information within a composite image but also reveals the correlation between contrasts.

The integration of concomitant, but different, physicochemical properties reflected in unique optical signals (Table I), strengthens the analytical capabilities of the nonlinear microscope. A simultaneous acquisition of these signals, therefore, reveals the structural, chemical, and functional properties of specimens that are otherwise inaccessible. Thus, by combining different modalities, the *multimodal* nonlinear microscope not only distinguishes the overlapping tissue components but also reveals spatial co-localization, capabilities that allow discovering functional relationships within the explored biological process. Table II presents the representative multimodal combinations that, in our experience, are effective for their associated applications.

**TABLE II.** Comparison of different multimodal applications of nonlinear microscopy.

Multimodal combination	Representative targets	Biological/clinical applications	Representative work
SHG + MPAF	Collagen, elastin, metabolic cofactors	Fibrosis detection, connective tissue imaging, tissue architecture (tumor-associated collagen) with metabolic status	28, 51
SHG + THG	Collagen fibrils, myelin, vesicles, cell membranes	Developmental biology, myelin mapping, tissue morphology	84–86
THG + MPAF	Lipid bodies, membranes, NAD(P)H/FAD	Lipid droplet dynamics, metabolic state in live cells, mitochondrial metabolism	40, 87–99
SHG + CRS (CARS/SRS)	Collagen, proteins, lipids, nucleic acids	Tumor margin detection, stromal–tumor interactions	90
MPAF + CRS	NAD(P)H/FAD, lipids, proteins, nucleic acids	Redox metabolism with molecular composition, cancer vs normal tissue discrimination, treatment response monitoring	52, 91, 92
SHG + MPAF + CRS	Collagen/ECM, NAD(P)H/FAD, lipids/proteins/nucleic acids	Integrated stromal architecture–meta-bolism–composition mapping; tumor microenvironment profiling, fibrosis staging	90
Full palette (SHG + THG + MPAF + CRS)	Collagen, elastin, NAD(P)H/FAD, lipids, proteins, nucleic acids	Integrated label-free histology, multimodal pathology, *in vivo* functional imaging	93

## III. ACQUISITION STRATEGIES IN MULTIMODAL LABEL-FREE NONLINEAR MICROSCOPY—SIMULTANEOUS VERSUS SEQUENTIAL MULTIPLEXED DETECTION

**FIG. 5.**
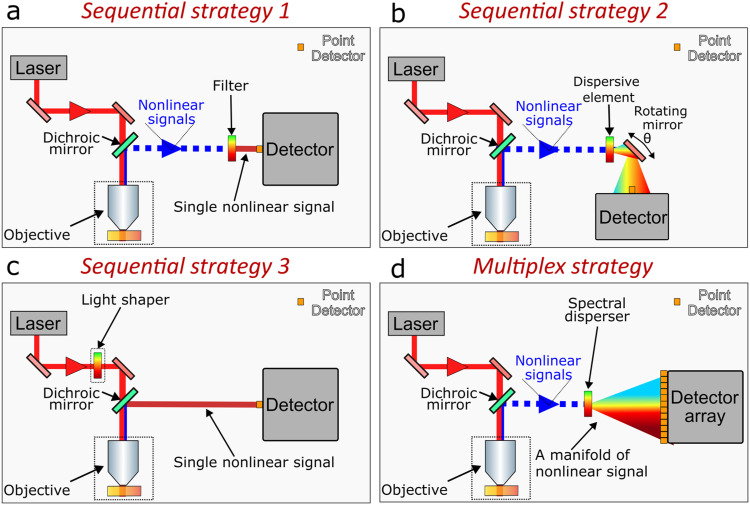
Acquisition strategies in multimodal label-free nonlinear microscopy. (a) Sequential acquisition by varying an optical filter, (b) sequential acquisition by scanning dispersed nonlinear signals with a mirror, (c) sequential acquisition by tailoring the excitation source, (d) multiplex acquisition, in which nonlinear signals are spectrally dispersed and acquired simultaneously.

Label-free multimodal nonlinear imaging refers to the acquisition of several nonlinear signals to derive contrast from different endogenous biomolecules, thereby delivering images with multiple channels that enable the unmixing and quantification of biochemical constituents. To acquire these contrasts, nonlinear microscopes can operate with either a sequential acquisition or a simultaneous acquisition multiplex strategy, each with unique advantages and limitations. Figure 5 depicts the simplified schematics of the sequential and simultaneous multiplex strategies often used. Below, we discuss these implementations for label-free multimodal imaging.

It is important to note that label-free nonlinear optical microscopy has historically relied on near-infrared excitation to minimize scattering and increase penetration depth, a set-up that negatively impacts spatial resolution. One way to address this problem is by shifting the driving fields into the visible range.^94,95^ Although at the expense of reduced penetration depth and increased risk of photodamage, this shift improves resolution by a factor determined by the excitation wavelength, while simultaneously enhancing signal generation by approaching electronic resonances. Additional strategies include adaptive optics to correct aberrations,^96–98^ super-resolution methods, such as multiphoton Stimulated Emission Depletion microscopy (STED) or Structural Illumination Microscopy (SIM),^99–101^ and computational approaches, including deconvolution or machine-learning-based restoration.^102,103^ These approaches complement acquisition strategies and are essential for the advancement of multimodal label-free microscopy. However, they lie beyond the scope of this Perspective.

### A. Sequential acquisition

Under a sequential acquisition approach, the nonlinear contrasts are acquired one at a time, either by collecting full images for each contrast, or by sequentially scanning different contrasts per pixel and iterating this process across the image to thus build a hyperspectral dataset. A key element of the sequential approach is the spectral discriminator—an optical, optomechanical, or electronic system dedicated to sifting through the manifold of nonlinear signals to isolate just one. The spectral discriminator further subdivides the sequential strategy.

**FIG. 6.**
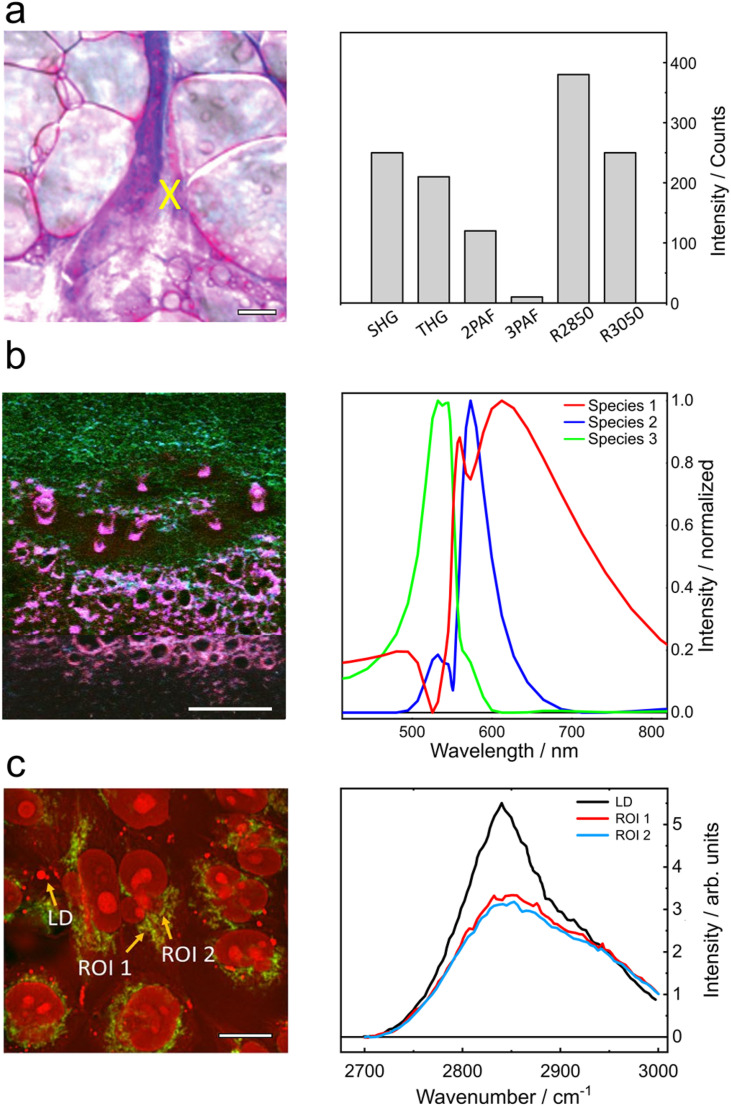
Label-free multimodal imaging using a sequential acquisition strategy. (a) “Optical histology” image obtained by sequentially acquiring SHG, THG, 2PAF, 3PAF, and CARS signals at 2850 and 3050 cm^−1^. The right panel shows a histogram of photon counts from the pixel marked with an “X.” (b) Hyperspectral images with MPAF and SHG contrast acquired by sequentially scanning the nonlinear signals onto a single-pixel detector. (c) Hyperspectral CARS imaging, sequentially acquired by tuning the instantaneous frequency of the excitation fields to probe different molecular vibrations. LD: lipid droplet; ROI: region of interest. Adapted from Refs. 16, 87, and 91.

The simplest, and in fact primeval,^13^ implementation of nonlinear microscopy uses an optical filter as a spectral discriminator, Fig. 5(a). In this branch of sequential scanning, the filter allows only a narrowband to reach the detector and the different contrasts are acquired by exchanging the filter, e.g., with a filter wheel. The left panel of Fig. 6(a) presents a composite image containing six nonlinear contrasts, each sequentially acquired using the aforementioned strategy.^87^ The right panel displays a histogram of the photon counts corresponding to the nonlinear signals at the pixel marked with an “X.”

A more advanced approach for sequential scanning employs an optomechanical setup comprising a diffraction grating and a fast-scanning element, such as a galvanometric mirror, to sequentially direct different spectral components onto a point detector [Fig. 5(b)]. An exemplary image acquired with this strategy is shown in Fig. 6(b), where the contrasts reflect the most prevalent chemical species of the sample. The pixels of this hyperspectral dataset contain 50 spectral points derived from MPAF and SHG signals.^16,104^

In another variation, the spectral discriminator acts not on the emitted nonlinear signals, but on the excitation fields, Fig. 5(c). By spectrally or temporally tailoring the excitation, either to isolate particular instantaneous frequency differences or to suppress competing interactions, it is possible to drive specific nonlinear signals. One such strategy, known as spectral focusing,^105–108^ is widely implemented in CRS, where the excitation fields are dispersed, and the signal of interest is probed by varying the instantaneous frequency difference between the pump and Stokes beams. Figure 6(c) shows a hyperspectral CARS image of the cells co-registered with MPAF.^91^ Alternatively, pulse shaping in the spectral domain, e.g., using acousto-optical modulators, allows for the selective transmission of specific excitation wavelengths, targeting different nonlinear interactions that enable the acquisition of nonlinear images with different contrasts.^109^

Another implementation for sequentially acquiring the nonlinear signals uses a bundle of optical fibers, whereby each transports a specific nonlinear signal.^84^ These fibers are identical, except on their length, which is varied to imprint a temporal delay between the nonlinear signals. With *a priori* knowledge of the temporal delay, synchronization of the laser clock with the readouts enables electronic multiplexing, thus supporting the subsequent separation of the nonlinear signals to produce multimodal maps.

### B. Simultaneous multiplex acquisition

**FIG. 7.**
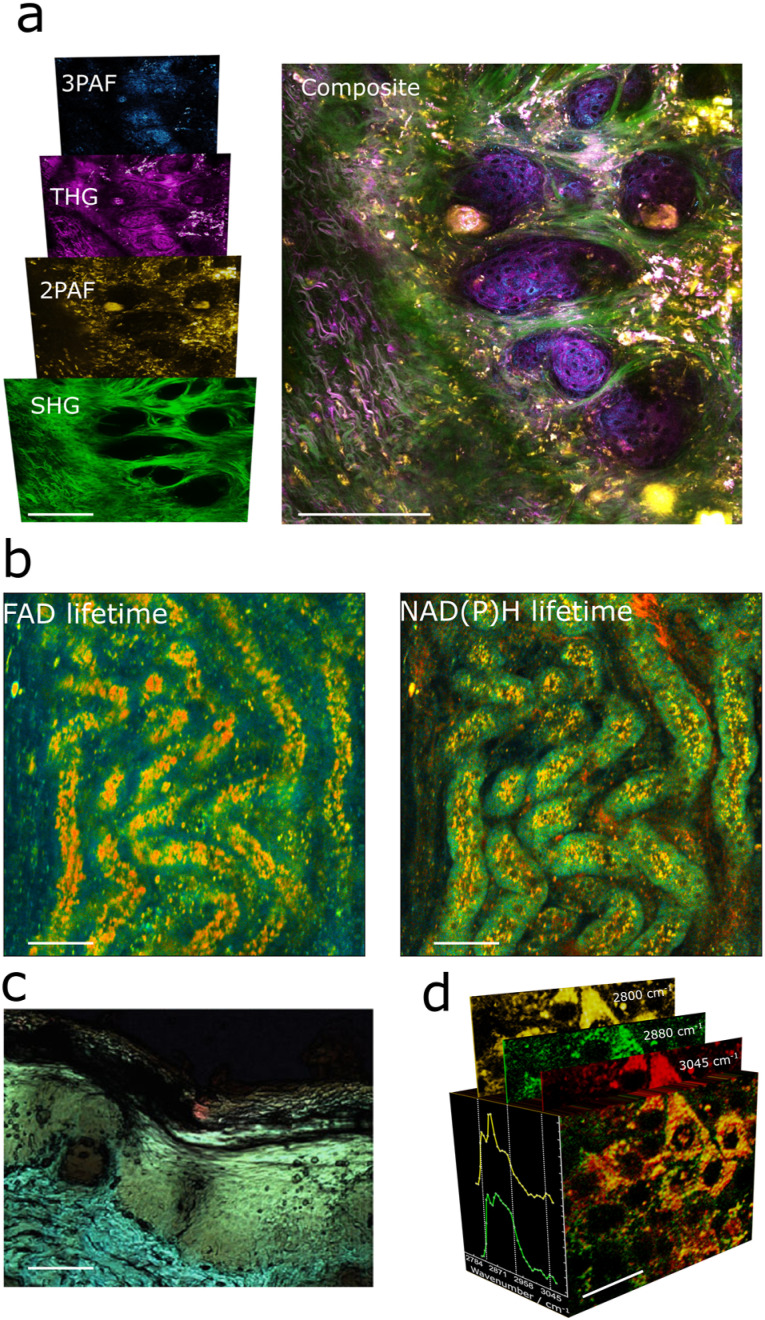
Label-free multimodal imaging using a multiplex acquisition strategy. (a) Exemplar SLAM image: the left panel shows concurrently acquired multiphoton contrasts, while the right panel depicts their composite. (b) Dual-channel FLIM images based on fluorescence decay rates of 2PAF (left) and 3PAF (right). (c) Hyperspectral CARS image of skin tissue. (d) Broadband SRS image of human hepatocytes. (a), (c), and (d) adapted from Refs. 110, 116, and 117. (b) Courtesy of Kevin Tan. Scale bars: (a) 100 *µ*m, (b) 50 *µ*m, (c) 100 *µ*m, and (d) 20 *µ*m.

The simultaneous multiplex strategy is conceptually simple: a spectral disperser separates the nonlinear signals and sends each to a single element of an array of detectors, thereby registering the contributions of each signal in parallel, Fig. 5(d). In this way, the multiplex strategy acquires hyperspectral data simultaneously in one single shot, such as from one single ultrafast optical pulse. The spectral disperser varies, targeting the nonlinear signal to be acquired. For multiphoton homodyne signals, i.e., where the signals emerge at a different optical mode relative to the excitation, the spectral disperser typically consists of an array of dichroic filters, each matched with a bandpass filter and a single-photon sensitive detector, such as a photomultiplier tube (PMT), which registers the intensity of each signal, enabling the co-registration of different contrasts. This technique is particularly amenable to the detection of harmonic signals and MPAF.^85,86,88^ Leveraging this approach, by generating widely coherent supercontinuum from pumped photonic crystal fibers and by compressing and strategically pulse-shaping the output, our group pioneered the concurrent acquisition of SHG, THG, 2PAF, and 3PAF.^89^ Figure 7(a) shows an image acquired using this strategy, which we branded as simultaneous label-free autofluorescence multi-harmonic (SLAM) microscopy.^110,111^ This setup also supports the resolution of fluorescence emission decays, thereby enabling fluorescence lifetime imaging (FLIM).^112–114^ FLIM adds additional analytical capabilities as it can disentangle different molecular species by means of their emission lifetimes, a magnitude that unequivocally identifies a given molecular system. Figure 7(b) illustrates the capabilities of FLIM, showing two simultaneously acquired images corresponding to different fluorescent species. This simultaneous acquisition strategy can also be extended to even more nonlinear modalities, including four-photon absorption, fifth-harmonic generation, and CRS.^92,115^

Naturally, multiplex implementations of nonlinear microscopy, most notably in broadband CARS microscopy, have employed grating-based spectrometers coupled with line detectors, such as a CCD or CMOS, as spectral dispersers. Owing to their high spectral resolution, spectral coverage, and parallel capabilities, these devices record full vibrational spectra per pixel in a single shot.^118–120^ Broadband hyperspectral data reveal the chemical landscape of the specimen, unleashing the potential of chemometric analysis and artificial intelligence to maximize information extraction from the sample. Figure 7(c) shows a hyperspectral CARS image from skin tissue acquired in this way.^59,116^

Recently, the growing popularity of SRS has spurred the development of broadband implementations. While high-bandwidth photodiode arrays are readily available, the need of SRS for electronic heterodyne amplification requires sophisticated multichannel electronics, leading to notable developments using resonant circuits for tuned amplifiers and multichannel lock-in detectors.^117,121–124^ Such implementations enhance sensitivity and enable simultaneous monitoring of multiple spectral channels. Figure 7(d) illustrates cells imaged using broadband SRS coupled with a multichannel lock-in amplifier, demonstrating the capability of this method to resolve biochemical contrasts through multimodal acquisition.

### C. On the challenges of multiplex approaches

The simultaneous detection of homodyne nonlinear optical signals faces several challenges. One notable challenge is spectral crosstalk,^125^ also known as crossover or bleed-through. Owing to the photophysics of endogenous biomolecules, some nonlinear contrasts are more intense than others. Spectral crosstalk then arises when a strong signal, e.g., SHG, bleeds into the detection channel of another contrast with scarce or no signal, say, THG. Consider, for example, muscles, tissues that exhibit intense SHG and a conspicuous THG, and brains, where there is an intense THG signal stemming from the myelin sheets with SHG nowhere to be found. Optical filters and dichroic mirrors effectively sift through the nonlinear spectrum, allowing only the nonlinear contrasts of interest into the detector and thus are the main barrier against crosstalk. For samples in which non-negligible crosstalk remains a problem, despite adequate hardware selection, post-processing helps reduce this effect. First, one must study the performance of the multimodal system for a given specimen, followed by the estimation of the contributions of spurious signals to a channel where its target contrast is absent. Upon estimation of this contribution, spectral crosstalk removal algorithms, most notably spectral linear unmixing,^126^ can be implemented.

A related challenge is the *imbalance* of signal intensities. While dependent on the sample, it is not rare that signals derived from two-photon processes, e.g., SHG and 2PAF, show stronger intensities than their counterparts derived from three- or higher-order photon interactions. As a result, the detectors targeting two-photon processes might be overwhelmed, whereas those from three- or higher-order interactions are photon-starved. This imbalance is manifested in the signal-to-noise ratio (SNR) of the images. In this context, the image quality from two-photon processes might be exquisite while that of the images from three-photon processes compromised. Note that reducing the excitation power is not a solution, for it will attenuate both signals, virtually annihilating any real contributions from the weak. The solution to the imbalance problem is once again tailoring the detection channels. For a given specimen, the experimentalist could determine which channels are overwhelmed with photons and which ones are starved. Then, the experimentalist may place neutral density filters on the detector of the intense contrast to prevent saturation, simultaneously allowing the collection of more photons on the weak channel. In tandem, the experimentalist may also rely on photodetectors with larger dynamic range in the intense channel, e.g., analog output PMTs, and detectors with lower dynamic range but higher sensitivity, e.g., photon counting PMTs, in the weak channel.

Finally, multiplex systems operating with a spectral disperser face the inevitable challenge of attenuation of already weak nonlinear signals by the dispersing optical element. Even more challenging are the slow acquisition rates imposed by currently available detector arrays. Although fast multichannel detectors lie on the horizon, at the time of writing, current technologies cannot deliver high-signal-to-noise ratio at video-rates. Thus, the photonics community is still awaiting such enabling technology.

In the meantime, the community can borrow denoising strategies from the computer vision field. By adopting emerging computational imaging approaches designed to recover high-quality images from photon-starved data, multiplex approaches—and label-free nonlinear imaging techniques, in general—could attain higher SNR through software-driven pathways.^127^

These challenges may explain why the multiplex strategy, despite its potential, is not the standard configuration in multimodal label-free nonlinear microscopy. Progress in detector technology, along with channel balancing strategies and detection schemes, will allow the multiplex configuration to reach its full potential, a potential that could enable this configuration to make a real impact on human health.

### D. On the limitations of sequential approaches and the benefits of simultaneous multiplex acquisition

Although the biochemical species summarized in Table I often occupy the same location within the interaction volume, leading to a concomitant emergence of the nonlinear signals, historical implementations of label-free nonlinear microscopy have relied on a sequential approach to capture these contrasts.^90,93,128,129^ While technically simpler than parallel detection, the sequential implementation, however, has three major drawbacks.

I. It requires scanning the sample multiple times to capture different signals. This means that if N contrasts are to be collected, the total acquisition time increases linearly by a factor of N. In certain applications, most notably those requiring rapid acquisition, such as in image guided surgery, this limitation becomes a roadblock. Therefore, increased acquisition times can hinder the practical application of nonlinear microscopes, while repeated scanning can have negative effects, particularly with living specimens, as prolonged laser radiation exposure might trigger phototoxic effects.
II. A sequential strategy prevents the strict spatial co-registration of the different nonlinear contrasts. Considering the natural dynamics of living specimens and the fact that a nonlinear microscope is prone to disturbances that are time-dependent—misalignment caused by mechanical vibrations, fluctuations of the optical source, and thermal drifts affecting the optics—sequential imaging can lead to spatial shifts between acquisitions. This is particularly true in situations where the nonlinear microscope is translated outside the optics laboratory, such as in the operating room or in the biological laboratory.
III. Sequential acquisition may result in asynchronous detection of signals that, when captured simultaneously, reflect coordinated aspects of a biological process. In other words, asynchronous detection can decouple temporally correlated signals, leading to false interpretations, which ultimately compromise the reliability of multimodal analyses.

**FIG. 8.**
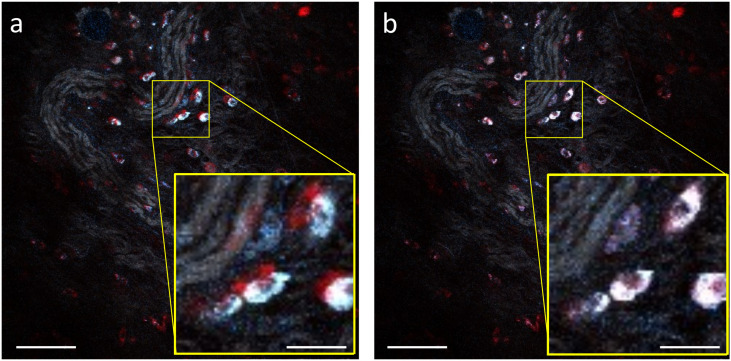
Spatial dynamics of *in vivo* cell and tissue autofluorescence. Comparison of label-free multimodal imaging using (a) a sequential and (b) a simultaneous multiplex acquisition strategy of cells in a dynamic *in vivo* tissue environment. In (a), the 2PAF channel was acquired 1 min after the 3PAF. Each image was registered within 2.5 s and consists of 500 × 500 pixels. The scale bars in (a) and (b) correspond to 100 *µ*m, while those in the insets correspond to 20 *µ*m.

We substantiate these arguments with data presented in Fig. 8, showing the results of *in vivo* imaging of a murine skin flap using SHG, THG, 2PAF, and 3PAF contrasts, employing both sequential and simultaneous multiplex strategies. In these images, we emphasize MPAF contrasts (2PAF shown in red and 3PAF in blue), while harmonic signals are displayed in grayscale. Figure 8(a) illustrates results from the sequential implementation, where MPAF contrasts from living cells after 1 min in their native environment (i.e., within a live specimen) appear displaced. Conversely, the simultaneously acquired multiplex strategy captures all structures and organisms, ensuring effective spatiotemporal co-registration of contrasts. Although it is difficult to ascertain the cause of the misregistration—animal breathing, cellular dynamics, or instrument misalignment—the results from the sequential strategy in Fig. 8(a) are clearly compromised. In contrast, Fig. 8(b) demonstrates well-matched 2PAF and 3PAF contrasts, confirming strict spatial co-registration of the signals. Finally, because in both tests we co-registered the harmonic signals with the 2PAF, the multiplex approach was two times faster than its sequential counterpart, further emphasizing the advantage and necessity of simultaneous multiplex acquisitions in label-free multimodal imaging.

Spatial misregistration has a negative impact that propagates downstream into the data processing pipeline. This is particularly true for one of the most powerful metrics for studying cellular metabolism, namely, the optical redox ratio (ORR). ORR represents the ratio between the fluorescence signals of oxidized FAD (fluorescent in its oxidized state) and reduced NADH (fluorescent in its reduced state). Because the emission spectra of NADH and its phosphorylated form NADPH are indistinguishable under steady-state measurements (see spectra Fig. 3), their combined signal is denoted as NAD(P)H. Although other definitions exist, here, we used $\text{ORR}\equiv \frac{2\text{PAF}}{2\text{PAF}+3\text{PAF}}\approx \frac{\text{FAD}}{\text{FAD}+\text{NAD}\left(P\right)H}$.^130–133^ The ORR leverages the opposite fluorescence dependencies on redox state of the metabolic cofactors FAD and NADH, thus providing a measure that distinguishes oxidative (high ORR) from reductive states (low ORR), thereby delivering a normalized measure of cellular metabolism.^134,135^ While the ORR is not a direct measurement of absolute concentrations, it is a relative metric that integrates the metabolic dynamics of NADH and FAD together into a redox-sensitive index.

We have observed that by exciting these metabolic cofactors with radiation centered around 1080–1140 nm, FAD and NAD(P)H autofluorescence intensity and lifetime can be detected via 2PAF and 3PAF, respectively. This simultaneous detection not only separates these metabolic cofactors but also enables the calculation of ORR on every pixel of the image, thereby providing an additional source of contrast that reveals the metabolic state of the specimen. Therefore, a reliable ORR image can only be obtained when the 2PAF and 3PAF signals are well co-registered, both spatially and temporally, while a lack of spatial or temporal alignment will naturally lead to artifacts that compromise the reliability of the data.

As discussed in Sec. II, the autofluorescence originate from specific molecules: NAD(P)H in its reduced form and FAD in its oxidized form. These metabolic cofactors highlight aspects of cellular metabolism and, when imaged simultaneously, reveal the redox balance in cells. In addition, mitochondria can be identified in the FAD channel, mainly because of the strong FAD fluorescence that reveals the characteristic filamentous morphology of this organelle.^136–138^ This instance shows how a spectroscopic signature, in this case the emission of oxidized FAD, maps back the well characterized structure of a cellular organelle.

**FIG. 9.**
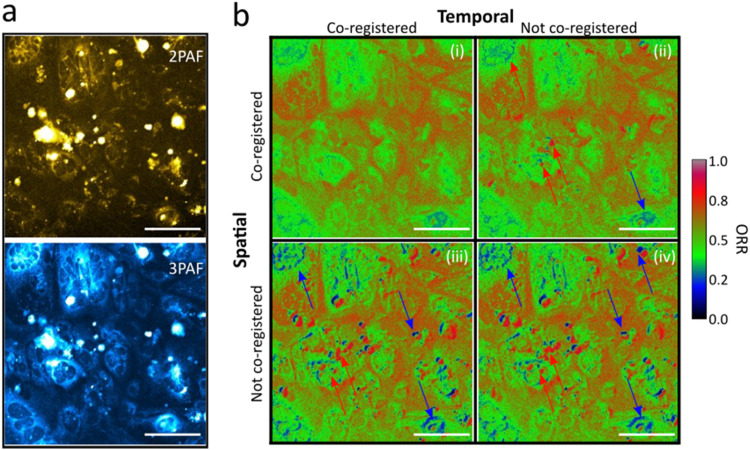
Impact of spatial and temporal misregistration in multimodal nonlinear microscopy. (a) Normal human hepatocytes (THLE-2) imaged using 2PAF and 3PAF. (b) Matrix of ORR images derived from Panel (a) under four different conditions: (i) strict spatial and temporal co-registration, (ii) static cells with slight optical misalignment (temporal shift), (iii) dynamic cells (spatial shift) with stable optics, and (iv) both cell motility (spatial) and slight optical (temporal) misalignment. To simulate sequential acquisition with minor optical drift, a 10-min delay was artificially introduced between the 2PAF (FAD) and 3PAF [NAD(P)H] channels during post-processing. To mimic spatial misregistration, NAD(P)H images were shifted by 4 pixels horizontally and 6 pixels vertically, corresponding to displacements of 2.8 and 4.2 *µ*m, respectively, relative to the FAD images. Imaging settings: 256 × 256 pixels. Scale bar: 100 *µ*m.

To illustrate the impact of misregistration, we used a multiplex strategy to map normal human hepatocytes (THLE-2), using 2PAF and 3PAF, to derive contrast [see Fig. 9(a)]. From this dataset, we calculated the ORR image, which, due to strict spatial and temporal co-registration, serves as a benchmark [see quadrant (i) in Fig. 9(b)]. We then explored three scenarios plausible during sequential acquisition. In all cases, the 2PAF contrast was acquired prior to the 3PAF. First, we assumed that the cells remained static, but the optical system was unstable due to laser misalignment caused by external disturbances, such as temperature-induced optical drifts or mechanical vibrations [see quadrant (ii) in Fig. 9(b)]. Next, we assumed that the optical setup was stable while the cells moved [see quadrant (iii) in Fig. 9(b)]. Finally, we assumed both active cell movement and laser system drift [see quadrant (iv) in Fig. 9(b)]. Note that in all scenarios except the benchmark, the 3PAF channel was slightly misaligned relative to the 2PAF channel, resulting in spatial misregistration that directly compromised the accuracy of the ORR calculation.

These results highlight that even subtle misalignments between contrasts can lead to misleading conclusions when the different signals are not strictly spatially and temporally co-registered. In our simulation, spatial displacements as small as 2.8 *µ*m and 10 min delays led to up 93.5% increase in the coefficient of variability of the ORR. Post-processing methods can help mitigate spatial mismatches, but they cannot fully restore the ground truth nor can they address the equally critical issue of temporal synchronization between channels.^139^

In addition to spatial misregistration, a sequential approach also leads to asynchronous detection of signals. This “temporal misalignment” fails to capture the correlations between signals, resulting in misinterpretation and reduced analytical reliability. Once again, the metabolic cofactors NAD(P)H and FAD help us demonstrate this issue. These molecules exhibit rapid temporal dynamics within cells and tissues, fluctuating within milliseconds in response to environmental stimuli and changes in cellular state.^140^ These changes are precisely what an ORR measurement is designed to capture. However, when 2PAF and 3PAF channels are acquired sequentially, the resulting ORR does not reflect the *instantaneous* metabolic state of the sample.

**FIG. 10.**
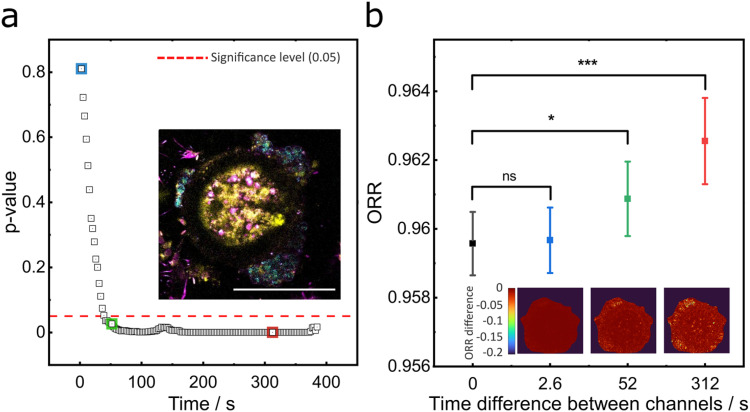
Temporal dynamics of cellular autofluorescence influence ORR measurements. (a) p-value analysis from pairwise comparisons of ORR maps calculated from time-lapse SLAM images of a live porcine oocyte. ORR was computed with varying temporal offsets between 2PAF and 3PAF channels. A statistically significant change (p < 0.05) in ORR appears when 2PAF is measured ≥40 s after 3PAF. The image inset shows a representative oocyte image used for this analysis. (b) Mean ORR values from conditions with increasing time differences between channel acquisitions: 0 s (black), 2.6 s (blue), 52 s (green), and 312 s (red). The inset images show pixelated ORR values in the oocyte. Significance levels: ns: nonsignificant, *p < 0.05, ***p < 0.001. Scale bar in (a): 100 *µ*m.

This effect is further demonstrated in Fig. 10, which shows how ORR changes with increasing time offset between channel acquisitions. We performed a time-lapse SLAM imaging experiment on a live porcine oocyte (a female germ cell). In this experiment, the 2PAF and 3PAF channels were acquired at varying delays to compute ORR maps. The mean ORR was calculated for each of the first 30 frames using a fixed 3PAF image from 0 s and 2PAF images at varying time points. The pairwise p-value analysis in Fig. 10(a) shows that even a 40-s offset results in a statistically significant difference in the ORR compared to the simultaneous acquisition baseline (p < 0.05). The mean ORR values plotted in Fig. 10(b) further highlight that asynchronous acquisition produces unreliable and misleading ORR readouts.

Manifestly, misregistration of contrasts is a critical issue in biomedical imaging, especially in time-dependent studies, such as monitoring cell viability, assessing tumor heterogeneity, or evaluating the responses of maladies to novel treatments. As our results demonstrate, sequential acquisition introduces significant risk of spatial and temporal artifacts, artifacts that can negatively impact the interpretation of results. In such applications, simultaneous multiplex acquisition is essential.

It is self-evident then that simultaneous multiplex detection overcomes the limitations of its sequential counterpart. By simultaneously acquiring the signals emerging from the same interaction volume, this multiplex strategy guarantees strict spatial co-registration of contrasts, assures synchronous detection of signals deriving from related dynamic biological processes,; and expedites acquisitions. Therefore, the simultaneous multiplex strategy delivers multimodal data that contain complex features and information that span across space, time, and modality.

## IV. ON THE INFORMATION CONTENT WITHIN LABEL-FREE MULTIMODAL DATA

A multimodal image consists of a three-dimensional matrix, whose rows (x) and columns (y) contain the scanned spatial positions of the sample, while each vector (z) orthogonal to the x–y plane stores the nonlinear signals at different spectral bands, i.e., the imaging modalities, see Figs. 6 and 7. Naturally, this can be further extended if a 3D spatial volume of image data is collected by the imaging system. For a given multimodal image, there are two main axes of relationships: inter-channel relationships and intra-channel relationships.

• Intra-channel relationships represent morphological patterns within each modality. These relationships include intensity, shape, and texture features.
• Inter-channel relationships refer to patterns manifested across channels, known as the co-localization of those signals.

While the ORR is a good example for inter-channel relationships, as it combines information across two channels that has shown to be informative for biological dynamics, many kinds of metrics can be calculated between different modalities to capture more of the complex relationships between channels. Because inter-channel relationships leverage the holistic information contained within the multimodal image, they are more informative than intra-channel relationships.

**FIG. 11.**
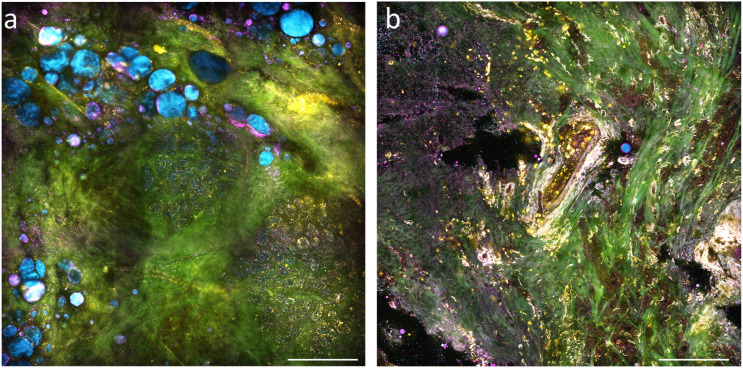
SLAM images of *ex vivo* (a) normal tissue and (b) cancer tissue from a mouse. Four imaging modalities are pseudocolored: THG (magenta), SHG (green), 3PAF (blue), and 2PAF (yellow). 3284 × 3284 pixels. The scale bars correspond to 100 *µ*m. Note how the SHG contrast highlights the extracellular matrix, which undergoes substantial remodeling during cancer pathogenesis. Similarly, observe the reduced lipid content in the cancerous tissue (blue, 3PAF), accompanied by an increase in 2PAF signal (yellow). Since 2PAF reports on FAD, this suggests elevated metabolic activity in the malignant regions.

Consider, for example, the multimodal images of normal tissue and cancer tissue shown in Fig. 11. The SHG signal (green) shows the structural organization of the tumor microenvironment, highlighting the collagen fibrils, whereas the 2PAF channel (yellow) shows the metabolic biomarker FAD. Given that in cancer pathogenesis, the morphological changes often obey chemical signals (from DNA expression), the relationship between these two channels could reveal the interplay between the morphological features of the specimen with its chemical composition—information that cannot be attained by relying on just one channel.

Thus, the more dimensions a multimodal imaging platform attains, the higher the information content extracted from specimens, an expansion that naturally escalates data complexity. These high-dimensional datasets are challenging for human interpretation, as they often exceed the capacity of visual inspection or intuitive comparison. Advanced computational approaches, particularly those grounded in machine learning (ML) and deep learning (DL), offer powerful tools for addressing this complexity.^31,32^ These methods excel at extracting diverse and subtle features, identifying distinctive patterns, and potentially uncovering unknown optical signatures that are not readily visible or interpretable by human observers.

Image-based profiling techniques using traditional ML approaches can extract a wide range of hand-engineered features from multichannel images, including intensity distributions, morphological characteristics, texture descriptors, and inter-channel correlations^141,142^ While these features can be highly informative, not all are relevant to a specific biological question. Therefore, feature selection techniques, such as recursive feature elimination (RFE), mutual information-based selection, or regularization methods, are critical for isolating the most biologically meaningful variables.^143–145^

Deep learning methods, particularly convolutional neural networks (CNNs), have emerged as highly effective alternatives to hand-crafted feature extraction.^146^ These models can learn hierarchical, abstract features directly from raw or minimally processed images, which often results in superior performance in downstream classification, segmentation, and regression tasks. DL approaches reduce reliance on extensive image preprocessing pipelines, which can sometimes introduce bias or lead to information loss, thereby making DL well-suited for multimodal image analysis, where retaining nuanced signals across channels is essential. In other words, DL works well for multimodal imaging because it can keep track of and learn from subtle differences in each channel, rather than losing or oversimplifying them. In addition, DL models tend to be more robust in the presence of noise, artifacts, or variable image quality, conditions frequently encountered in label-free nonlinear imaging of biological specimens.^147^

**FIG. 12.**
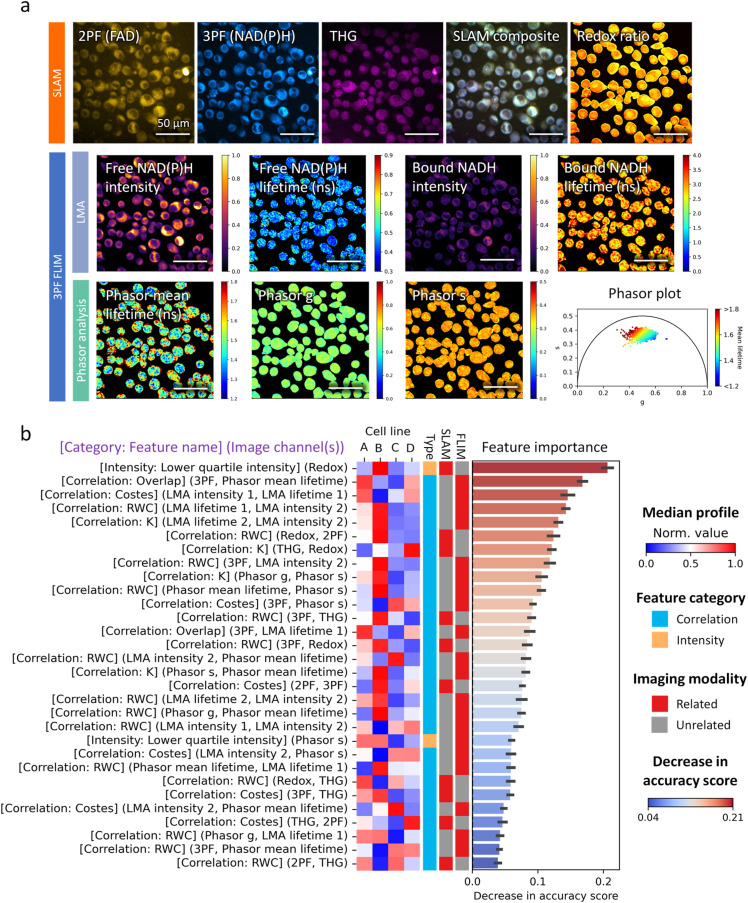
Biopharmaceutical CHO cell characterization using SLAM and 3PAF FLIM. (a) SLAM and FLIM images of a single field-of-view of CHO cell samples. The optical redox ratio was computed from the 2PAF (FAD) and 3PAF [NAD(P)H] channels. NAD(P)H lifetimes were estimated using both phasor analysis and biexponential fitting via the Levenberg–Marquardt algorithm. The phase plot (bottom right) displays the phasor coordinates (g, s) and mean lifetime of cell pixels in the image. (b) Permutation feature importance ranking of the top 30 features for CHO cell line classification. Cellular features were extracted using CellProfiler.^142^ The associated imaging modalities, feature categories, and median feature profiles across different cell lines are shown. Adapted from Ref. 151.

To illustrate these concepts, we present Chinese hamster ovary (CHO) cell lines with distinct production phenotypes in Fig. 12(a). To comprehensively characterize the single-cell morphology and molecular features of these cells, we measured the THG, 2PAF, and 3PAF signals, along with emission lifetime data from the 3PAF channel. This multimodal configuration delivered 1480 features per cell. These features spanned multiple categories, which include intensity, morphology, texture, radial distribution, granularity, and inter-channel correlation, thereby capturing the rich spatial and biochemical information embedded in the multimodal imaging data. To identify the most informative features for cell line classification, we applied a data-driven approach combining RFE with supervised machine learning. Notably, correlation-based features, i.e., quantifying the spatial co-occurrence and co-localization of intensities between imaging modalities, consistently ranked among the most important features for the CHO cell line across all cell passages studied, Fig. 12(b). These features have previously been employed to elucidate protein–protein interactions, molecular co-localization, and intracellular signaling networks.^148–150^ Therefore, Fig. 12 underscores how crucial inter-modality correlation is for distinguishing complex biological features, emphasizing not only the strength of co-localization analysis but also the added value that multimodal imaging brings in uncovering mechanistic insights into biological systems.

The integration of ML and DL with multimodal imaging has already demonstrated significant success in a variety of applications. These include the discovery of novel cancer biomarkers through integrative imaging phenotypes,^152,153^ automated classification of cell states or tissue types,^151,154^ drug screening and distribution analysis,^155^ and uncovering mechanisms of disease progression from complex image datasets.^156^ Looking ahead, the continued convergence of multimodal imaging and artificial intelligence promises not only to enhance analytical capabilities but also to shift the paradigm from descriptive to predictive and mechanistic biology. By uncovering patterns and high-dimensional relationships, label-free multimodal imaging can accelerate hypothesis generation, improve reproducibility, and enable a more holistic understanding of biological systems.

Furthermore, DL has established the concept of computational labeling in linear label-free imaging, whereby images formed with a given optical signal are translated into maps of phenomenologically different contrasts.^157^ Notable examples include the translation of quantitative phase images into “synthetic” fluorescence-labels counterparts^158^ and the decoding of 3D refractive index tomograms into multiple fluorescence-equivalent channels.^159^ These studies provide an important service to the community working on multimodal label-free nonlinear microscopy, as they pave the way for adapting such developments in this field: *networks could now be trained to infer nonlinear contrasts that are difficult or impractical to obtain directly from those more readily accessible*. Collectively, these works highlight how AI could not only support label-free multimodal nonlinear microscopy but also enhance it.

Beyond virtual labeling, AI could also be applied to enhance the instrument itself. For instance, the label-free nonlinear microscope could leverage DL-based denoising and super-resolution frameworks to improve both the signal-to-noise ratio^127,160^ and spatiotemporal resolution,^161,162^ an improvement that would enable more reliable quantification and visualization of biological activity. The label-free nonlinear optical microscope could also benefit from other recent studies that have focused on self-driving microscopy platforms, whereby the instruments integrate real-time AI analysis with multimodal acquisition.^163–165^ This integration would allow the microscope to adaptively adjust illumination, scanning trajectories, and modality switching to maximize information content while minimizing photodamage. These developments could transform this technology into a predictive and mechanistically interpretable tool for biomedical discovery.

## V. CONCLUSIONS AND PERSPECTIVE

Label-free nonlinear microscopy has maturated as a versatile tool for the biomedical sciences, with demonstrated potential since its inception in the early 1980s. This versatility mainly stems from the capacity of this set of imaging modalities to map cells and tissues with signals emerging from endogenous biomolecules and microstructures, thereby preserving the physiological viability and functionality of cells and specimens. Technological advancements—especially improvements in detectors, instrumentation hardware, optical filters, and most notably the advent of compact, stable, and affordable femtosecond laser sources—have pushed this technology into clinical settings and biological laboratories. Testament to the versatility of this technology is the wide range of applications it supports, applications that range from biopharmaceuticals and systems biology to fundamental research on disease pathogenesis to intraoperative image guidance and surgical decision making.

At the time of writing, the most sophisticated label-free nonlinear microscopes are multimodal. They capitalize on the heterogeneity of biological specimens, which mediate not one but many nonlinear signals to derive contrast. Because these nonlinear signals originate from specific physicochemical and microstructural properties of specimens, they each inform about unique features of the sample, either revealing the architectural or chemical properties, or in some cases, both. Multimodal acquisition then affords a robust contrast palette with complementary signals that enable not only the visual separation of constituents but also their quantification and their corresponding relationships. These superior analytical capabilities can be complemented by deploying statistical analyses that reveal the correlation between the different nonlinear contrasts, which help to determine how different contrasts—or morphofunctional properties of the sample—evolve or interact. Furthermore, the multidimensional nature of this imaging approach perfectly resonates with artificial intelligence: By feeding multimodal datasets to machine learning or deep learning networks, artificial intelligence can extract patterns and subtle features that are often imperceptible to human observers.

While two multiple acquisition strategies, sequential and simultaneous, exist for acquiring multimodal datasets, we have shown that a sequential acquisition approach suffers from increased imaging time, spatial misregistration due to sample dynamics and system instabilities, and temporal desynchronization of biologically correlated signals. Although these factors do not limit the practical utility of a sequential strategy, they may compromise the reliability of its data, a deficiency that has strong implications, especially in dynamic or clinical settings. Because simultaneous multiplex detection overcomes all the limitations of its sequential counterpart, it then appears as the obvious strategy for multimodal imaging. Yet, it is not widely implemented and used. The reason most likely lies on the difficulties related to channel scalability. While the multiplex data from label-free nonlinear microscopes, such as those shown in Figs. 7(a) and 7(b), uses a manifold of contrasts that afford multimodal imaging, they still correspond to bands of a full spectrum, disregarding a wealth of information that “is already there.” This spectral-under sampling compromises the analytical capabilities of the nonlinear microscope because four or five bands do not fully reconstruct a spectrum. While it is true that some multiplex implementations are capable of acquiring full spectra per pixel, these approaches are still slow, and do not reach video-rate imaging for capturing biological processes.

Still, even after refining acquisition strategies, label-free nonlinear multimodal microscopy remains constrained by the weak nature of its signals: endogenous molecules are weakly couple to the driving fields—we refer the interested reader to the extensive literature on how cross sections and nonlinear susceptibilities influence nonlinear signals.^6,41,167^ While pulse shaping and advanced detection strategies can partially compensate these physical limits, they fail to overcome them. This fundamental weakness accentuates the challenge of scaling multimodal detection and highlights the need for new approaches, approaches that lie beyond optics and photonics.

We therefore identify a technological gap that cannot be met by the optics and photonics communities alone, but rather through advances in electronic instrumentation. Although current biomedical applications remain limited, the fast-paced evolution of these technologies makes the future bright for the label-free multimodal nonlinear microscope, a future in which we anticipate this instrument to transition from a benchmark technology into a clinical standard of care.

## Data Availability

The data that support the findings of this study are available from the corresponding author upon reasonable request.
